# Editorial: Molecular underpinnings of genetic and rare diseases: from diagnostic tools to therapeutic approaches

**DOI:** 10.3389/fmolb.2025.1722468

**Published:** 2025-11-10

**Authors:** Abhilash Kumar Tripathi

**Affiliations:** Fujifilm Diosynth Biotechnologies Texas LLC, College Station, TX, United States

**Keywords:** rare disease, diagnosis, genetic variants, precision medicine, genetic diseases

Rare diseases are one of the most complex puzzles in medicine because they sit at the intersection of complexity and precision. The scarcity and molecular diversity of rare diseases make them challenging to diagnose and treat. For example, molecular diversity such as small nucleotide changes in genetic sequences, genetic rearrangements, or subtle changes in regulatory mechanisms can severely alter biology, and result in severe outcomes. The studies included in this Research Topic i.e., Molecular Underpinnings of Genetic and Rare Diseases, shows how much technological, computational, and technological progress has been made in the field of rare disease research. The studies published in this Research Topic show how we are moving from simply identifying variants of interest toward truly understanding how such variants impact proteins, cells, and ultimately a patients’ wellbeing.

For all researchers working in the field of rare disease diagnosis, structural rearrangements are extremely frustrating since they often present the risk of being undetected and overlooked. In Gallardo et al., the authors reveal how usage of CRISPR/Cas9-based enrichment in tandem with long-read nanopore sequencing can elucidate the location, size, and orientation of the variant in PAH gene implicated in Phenylketonuria (PKU). Gallardo et al. discovered an ∼18 kb tandem duplication between exons 1 and 3 of the PAH gene. Using nCATS and structural modeling, the authors revealed a mild but relevant alteration in PAH enzymatic function. One of the most outstanding parts of this research was the structural modeling work that suggested that the duplicated exon instead of inactivating the enzyme, it perturbs PAH enzyme interaction with cofactors that results in the onset of disease but it is not severe. The work by Gallardo et al. reminds us that pathogenicity is sometimes due to subtle biochemical changes and it is not always binary in nature.

Additionally, often the most trickiest aspect of clinical genetics is understanding whether a variant is actually matters and is pathogenic. Two studies in this Research Topic aim to tackle this question from different angles. Gajardo et al. examined the IFT140 missense variants associated with Mainzer–Saldino syndrome (MCC) using ΔΔG-based protein stability predictions. Through comparison against known pathogenic and benign mutations in MCC, Gajardo et al. proposed a quantitative threshold (−1.3 kcal/mol) to help classify uncertain variants. The authors adopted a practical approach by not only relying on abstract modeling but they tested the cutoff and indicated it could reclassify an actual clinical case.

On the other hand, Kušíková et al. elucidated the genotype spectrum in PIGQ variant observed in multiple congenital anomalies-hypotonia-seizures syndrome 4 (MCAHS4). Using cell-based assays, Kušíková et al. identified two novel PIGQ variants causing MCAHS4 and showed that p.L457R variant disrupts GPI-anchored protein expression. Additionally, the study used AI-assisted facial phenotype analysis (GestaltMatcher) to demonstrate patient clustering with other PIGQ-related cases. This study by Kušíková et al. is very exciting as it shows how computational biology, clinical genetics, and digital phenotyping can be used together in a productive for diagnosis of rare diseases.

Although genetics is unarguably the entry point in rare disease research, it does not present the complete picture since there are several other associated factors that drive the progression of a rare disease. For instance, Passaro et al. presented a comprehensive review on pathophysiological mechanisms of keratoconus with an emphasis on oxidative stress, and inflammation as drivers of disease. This review article is a great reminder that genetic along with acquired mechanisms often overlap and that researchers working in the field of rare diseases should focus on cellular stress pathways in addition to identifying variants in the DNA sequence.

The paper by Abozaid et al. highlighted the policymaking framework, and resource allocation for rare diseases which is something equally important but often overlooked. Working within the Saudi healthcare framework, Abozaid et al. proposed criteria that for policymaking and funding. They emphasized that prevalence along with disease severity and unmet medical need should drive policymaking, and resource allocation. As scientists, we sometimes forget how definitions of a disease influence what gets studied and funded. The work by Abozaid et al. illustrates that even the language used to describe rarity has real-world implications for patients seeking diagnosis and treatment.

Looking at all these contributions, a clear sequence emerges in the field of rare disease research. Undoubtably, the first step is discovery that includes discovering of disease causing variants. The second step is prediction where computational models are used to estimate molecular effects of the variants identified. This is followed by validation studies using cell-based assays or structural modeling to confirm impact of the disease, and phenotype integration to understand molecular and clinical signatures. And ultimately, policy frameworks and funding to ensure that these findings reach patients in need.

The usage of technologies such as long-read sequencing, stability modeling, functional assays, and phenotypic AI in papers published under this Research Topic demonstrates that rare disease research requires a multi-layered toolkit. However, there are still hurdles such as the labor-intensive nature of functional testing, varying of predictive threshold by gene, and population biases reflected in the usage of machine learning models. But overall, the field if rare disease is maturing, and our ability to connect discovery science to the lived experience of patients with rare disease is a milestone worth celebrating.

In conclusion, if there is a single theme running through all these papers, it’s that precision medicine is no longer theoretical rather it’s happening right now. Once considered obscure, Rare diseases, are now at the forefront of the future of personalized medicine, and that’s exactly the kind of progress we need.

